# Posterior interosseous nerve lesion due to lipoma. Review of the literature and rare case presentation

**DOI:** 10.2478/raon-2024-0041

**Published:** 2024-10-04

**Authors:** Bojan Rojc, Peter Golob

**Affiliations:** General Hospital Izola, Izola, Slovenia; Faculty of Mathematics, Natural Sciences and Information Technologies, University of Primorska, Koper, Slovenia

**Keywords:** posterior interosseus nerve, lipoma, compression, acute

## Abstract

**Background:**

Posterior interosseous nerve lesion is a rare mononeuropathy of the upper limb. Atraumatic posterior interosseous nerve lesions are commonly caused by lipomas of the forearm, manifesting as slow-progressing wrist and finger drop.

**Patients and methods:**

In this review and case report study, we present a systematic review of the literature for patients presenting with posterior interosseous palsy due to lipomas and a rare case of patient with acute posterior interosseous nerve lesion caused by a lipoma. Our primary interest was in the timing of clinical presentation. For the review process, we followed the Preferred Reporting Items for Systematic Reviews and Meta-Analysis guidelines.

**Results:**

After reviewing the literature, we identified thirty patients with posterior interosseous nerve lesions caused by lipomas. In 28 patients, the symptoms presented progressively, ranging from 1 month to a maximum of 240 months. We found only one case of a patient with acute presentation and another patient with acute worsening of chronic weakness due to trauma.

**Conclusions:**

Atraumatic posterior interosseous nerve lesions are frequently secondary to forearm lipomas. In the majority of cases, the symptoms will develope progressively. However, in this study, we also report a rare case of a patient presenting with acute posterior interosseous nerve lesion due to a lipoma.

## Introduction

Upper arm mononeuropathies are common pathologies, particularly entrapment neuropathies affecting median and ulnar nerves.^1^ While radial nerve lesions are less frequent, but they still occur, often presenting as radial neuropathy at spiral groove due to extrinsic compression. In rare cases, a lesion of the radial nerve can occur at the forearm.^1^

At the lateral epicondyle level, the radial nerve bifurcates into two branches: the superficial radial sensory nerve and the deep radial motor branch. The motor branch enters the supinator muscle beneath the Arcade of Frohse, where it is known as the Posterior Interosseus Nerve (PIN). PIN is almost exclusively a motor nerve providing innervation to the extensor carpi ulnaris, extensor digitorum communis, extensor digiti quinti, abductor pollicis longus, extensor pollicis longus, extensor pollicis brevis, and extensor indicis proprius muscle.^2^

Nontraumatic PIN neuropathy at the elbow is a rare condition with an annual incidence of 0.003%.^1,3^ However, some confusion and controversies exist regarding the nomenclature of nontraumatic PIN neuropathy in the elbow. Radial tunnel syndrome (RTS) is defined as a compressive neuropathy of the PIN, causing pain and tenderness 3–5 cm distal to the lateral epicondyle, without motor signs.^4,5^ Despite the belief that RTS results from PIN compression, most cases do not show abnormalities on medical imaging or electrodiagnostic testing.^6^ On the other hand, PIN lesion or PIN syndrome (PINS) presents most often with slow onset weakness of the muscles innervated by the PIN, without sensory findings.^2^ PIN lesion can be further categorized in compressive and non-compressive (neuralgic amyotrophy, hourglass-like fascicular constriction).^7^

There are five potential sites of intrinsic compression of the PIN at the proximal forearm:

1. fibrous bands of tissue anterior to the radiocapitellar joint between the brachialis and brachioradialis muscle;
2. the recurrent radial vessels that fan out across the PIN (“leash of Henry”);
3. the leading edge of the extensor carpi radialis brevis muscle;
4. arcade of Frohse;
5. and the distal edge of the supinator muscle.^1,7,8,9^

Occupations involving repetitive pronation and supination movements are considered to be a risk factor for PINS.^7^ Extrinsic compression of the PIN can result from various pathologies, with as many as 30 different pathologies described.^7^ Among these, lipoma is the most reported pathology causing extrinsic compression of PIN.^9,10,11^

Lipomas are slow-growing benign tumours composed of adipose cells encapsulated by a thin layer of fibrous tissue.^12^ They can be classified based on their anatomical site into dermal, subcutaneous and sub-fascial lipomas, or tumours directly related to muscle, bone, synovium or nerve.^13^ In the context of PINS, parosteal, intermuscular and intramuscular types of lipomas have been most often reported. Due to their slow growth, lipomas predominantly cause progressive PIN palsy.

## Patients and methods

For the review process, we adhered to the Preferred Reporting Items for Systematic Reviews and Meta-Analysis (PRISMA) guidelines. The authors conducted searches on PubMed, MEDLINE and Google scholar using keywords: posterior interosseus nerve, palsy, and lipoma. Through this procedure, we identified 47 studies. Only full-text articles in English or translated versions were accepted for further screening. Our search encompassed studies published up to February 2024. Subsequently, we analysed these studies to identify those reporting patients with PINS due to lipoma. We included only studies where the manifestation of symptoms (whether progressive or acute) was clearly stated. Ultimately, we reviewed 24 studies, which collectively reported on 30 patients (Table 1).

**TABLE 1.** Twenty-four studies reported 30 patients with posterior interosseous palsy due to lipomas

**Patient**	**Ref**	**Year**	**Age (years)**	**Sex**	**Onset**	**Duration (months)**	**Lipoma**	**Recovery**
1	Vikas^14^	2020	54	F	Progressive	5	Parosteal	Complete
2	Allagui^15^	2014	28	F	Progressive	6	Intramuscular	Complete
3	Maldonado^16^	2017	78	M	Progressive	8	Parosteal	Incomplete
4	Maldonado^16^	2017	65	F	Progressive	30	Parosteal	Incomplete
5	Yamamoto^17^	2016	60	F	No symptoms	?	Intermuscular	Complete
6	Rishab^18^	2021	47	M	No symptoms	?	Intramuscular	Complete
7	Flores Robles^19^	2017	40	M	Progressive	1	?	Complete
8	Salama^20^	2010	83	F	Acute Trauma		Parosteal	Complete
9	Saaiq^21^	2017	53	M	Progressive	7	Parosteal	Complete
10	Patel^22^	2018	66	M	Progressive	4	Intraneural	Near Complete
11	Murphy^23^	2009	58	M	Progressive	<1	Intramuscular	Near Complete
12	Nishida^24^	1998	60	F	Progressive	2	Parosteal	Complete
13	Nishida^24^	1998	61	F	Progressive	?	Parosteal	Complete
14	Ganapathy^25^	2006	54	M	Progressive	144	Intramuscular	Near Complete
15	Colasanti^26^	2016	59	F	Progressive	6	Intermuscular	Complete
16	Avram^27^	2004	69	M	Progressive	4	Parosteal	Partial
17	Hamdi^28^	2010	59	M	Progressive	2	Parosteal	Complete
18	Matsuo^29^	2007	60	M	Progressive	240	Intraneural	Poor
19	Seki^30^	2012	67	F	Progressive	2	Parosteal	Complete
20	Eralp^31^	2006	45	M	Progressive	?	?	Complete
21	Fitzgerald^32^	2002	71	F	Progressive	1	?	No recovery
22	Fitzgerald^32^	2002	62	M	Progressive	2	?	Complete
23	Fitzgerald^32^	2002	64	M	Progressive	3	?	Complete
24	Fitzgerald^32^	2002	68	F	Progressive	5	?	Complete
25	Fitzgerald^32^	2002	63	F	Progressive	2	?	No recovery
26	Narayan^33^	2016	46	F	Progressive	6	Parosteal	?
27	El Hyaoui^34^	2014	68	F	Progressive	14	Parosteal	?
28	Borman^35^	2010	69	F	Progressive	6	Parosteal	Partial
29	Richmond^36^	1953	62	M	Progressive	3	Intramuscular	Near Complete
30	Bugnicourt^37^	2009	48	M	Acute		?	?

F = female; M = male; ? = no data

### Case

**FIGURE 1.**
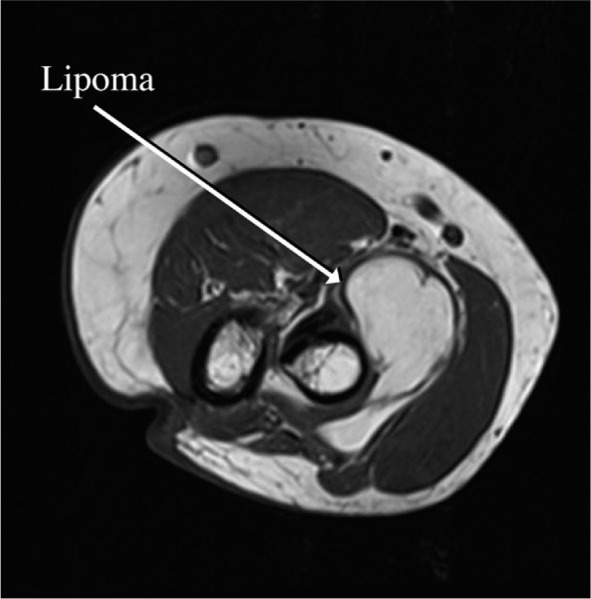
MRI of left elbow.

A 68-year-old female patient presented to the emergency room of our hospital with weakness of left wrist and fingers extension. In her past medical history, she reported having diabetes and arterial hypertension. The current symptoms had started 3 days prior to her visit. The patient mentioned strenuous work involving her arms due to cleaning, which included repetitive pronation and supination movements. A few hours after this activity she suddenly noticed weakness extending her fingers, without experiencing pain or paraesthesia. Clinical examination revealed weakness in left wrist extension accompanied by slight radial deviation (Muscle Power Scale - MRC 3) and more pronounced weakness in left fingers extension (MRC 2), the strength of other muscle groups of the left arm was preserved (MRC 5). There were no sensory deficits. To rule out possible brain vascular lesions brain computer tomography (CT) and CT angiography of cerebral arteries were performed, but the imaging did not show acute stroke or arterial narrowing. Based on these findings and patient's history a clinical diagnosis of left PIN palsy due to intrinsic entrapment caused by repetitive movements was made. Electromyography (EMG) performed a week after symptoms onset confirmed PIN lesion, showing denervation with fibrillations potentials and positive sharp waves (on scale 2 out of 3) and motor unit potential reduction in PIN innervated extensor indicis proprius muscle, brachioradial muscle did not show any signs of denervation. The superficial sensory radial nerve conduction study was normal. At the follow-up visit after 4 weeks, there was no improvement of muscle strength. Consequently, we decided to perform a magnetic resonance imaging (MRI) scan of the left elbow, which revealed a 50 x 40 x 25 mm lipoma as a probable cause of nerve compression (Figure 1). The patient included in this study provided written informed consent for the publication of anonymized data in accordance with the declaration of Helsinki.

**FIGURE 2.**
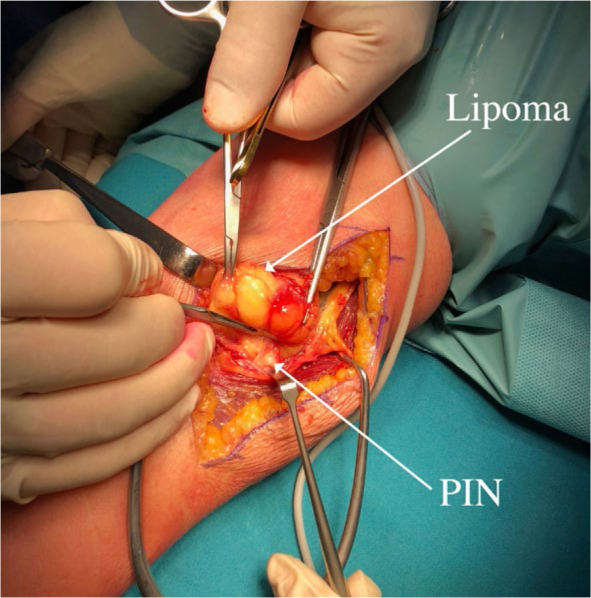
Lateral dissection along the common radial nerve and posterior interosseus nerve (PIN).

**FIGURE 3.**
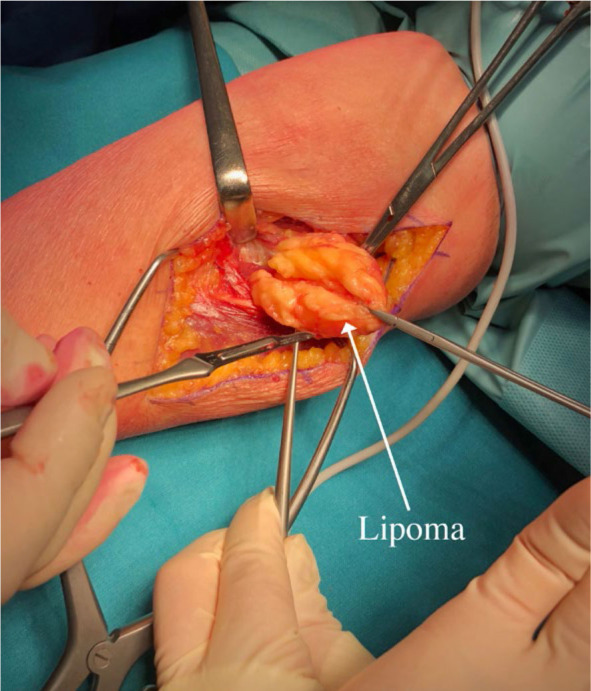
Medial dissection away from posterior interosseus nerve (PIN).

**FIGURE 4.**
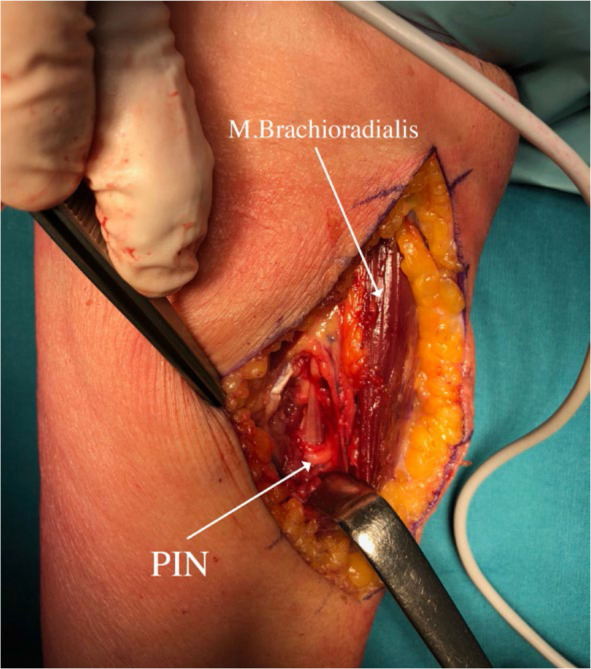
Local situation after lipoma removal PIN = posterior interosseus nerve.

Subsequently, after 2 months, surgery was performed on the left forearm to remove the lipoma. During the surgical procedure, the patient was placed in supine position arm resting on a side table. Our dissection began above the elbow, in the groove between brachialis/biceps muscles medially and brachioradialis muscle laterally, aiming to expose common radial nerve. We followed the nerve distally into the proximal forearm, where the brachioradialis muscle was retracted laterally, providing exposure to the tumorous mass. The tumour was situated between cutaneous branches on its lateral and anterior side and PIN on its medial and posterior side. Cutaneous branches were then dissected off the tumour and retracted laterally allowing for further dissection going from lateral to medial and anterior to posterior. This gave us exposure to the PIN lying below the tumour tethered to its pseudo-capsule at the point of PIN entry into the supinator muscle (Figure 2). We released the PIN and continued resection towards the neck of the radius, where the tumor reached into the depth and terminated. The tumour was removed en-bloc and sent to histopathological examination, which confirmed the diagnosis of lipoma (Figure 3 and 4).

At the follow-up visit after 11 months the patient demonstrated substantial clinical improvement. Remarkably after 24 months there was no weakness in left finger and wrist extension.

To our knowledge, this case represents a rare instance of acute nontraumatic PINS caused by a lipoma.

## Results

We identified 30 patients with PIN palsy caused by lipoma compression. The median age of these patients was 59.6 years, ranging from 28 to 83 years. Among the 30 patients 28 experienced a progressive build-up of symptoms, which varied from less than a month to a maximum 240 months. Only one patient was reported to have an acute manifestation of symptoms, while another patient had acute worsening of chronic weakness after trauma and immobilisation. Both female and male patients were equally represented, 15 females and 15 males. The most predominant type of lipoma observed was parosteal occurring in 13 patients. In most cases, the recovery was complete or near complete (Table 1).

## Discussion

Entrapment neuropathies of upper arm are common conditions, primary manifesting as carpal tunnel or cubital tunnel syndrome.^3^ In contrast, nontraumatic PIN palsy is a rare condition, with prevalence of only around 0.003 %.^1,3,38^ Patients with PIN palsy present with motor symptoms due to weakness of PIN innervated muscles in the forearm.^1^ Clinically, it can resemble the lesion of radial nerve at the spiral groove. Both conditions present with wrist and finger drop, sparing the elbow extension. However, two important differences set them apart. In a PIN lesion, the muscles innervated above the take-off of the PIN are spared, allowing these patients to weakly extend the wrist with radial deviation, and there are no sensory findings.

Atraumatic PIN lesions most commonly result from compressive pathology at the level of upper forearm.^2^ There are 5 well-known sites at the elbow that can cause intrinsic compression - entrapment of the PIN, with repetitive pronation and supination movements being a well-established predisposing factor.^1,7^ Conversely extrinsic compression of the PIN is most often due to lipoma.^9,10,11^ In both cases a slow progressive build-up of symptoms is expected.^2^

Our patient presented with acute PIN lesion caused by a parosteal lipoma. She also reported repetitive pronation and supination movements in the preceding days. In the reviewed literature, we found only one case of acute PIN lesion caused by lipoma, mimicking stroke. In that case, the symptoms started suddenly, and no predisposing activities were reported.^37^ Another case involved a 83-year-old woman who had a combination of chronic PIN lesion caused by a lipoma and an acute worsening after forearm immobilisation due to distal radius fracture.^20^ We suggest that the acute presentation in our patient is most likely due to nerve traction caused by lipoma movement during repetitive arm pronation and supination. Based on EMG findings, showing denervation a week after symptoms onset, we can assume that there was some longstanding axonal nerve injury due to lipoma growth. We suppose that the acute manifestation of symptoms was caused by nerve demyelination block. This would also explain the good recovery. Unfortunately, we do not have motor conduction studies to prove this suggestion.

The recommended treatment for patients with PIN lesion due to lipoma is surgical excision.^1,7,39^ Fortunately, most patients recover well and the symptom duration serves as a predictor for good recovery.^39^ After surgery and removal of the lipoma, our patients showed complete restitution of function after 24 months.

PIN lesion due to lipoma is rarely encountered in clinical practice. The most common clinical scenario involves progressive weakness of wrist and finger extension, accompanied by a palpable mass at the proximal forearm. As presented in our review, the acute presentations are very rare. Nevertheless, it is advisable to perform imaging studies of elbow in all patients with PIN lesion, as a substantial proportion of cases are secondary to expansive masses surrounding the nerve. We propose as imaging method of choice nerve ultrasonography^8^ or MR imaging. This recommendation holds true, especially considering the good prognosis associated with surgical removal.
